# Targeting Vascular Aging in Progeria by mTOR Inhibitors from Multi-Omics Screening

**DOI:** 10.1093/geroni/igaf122.3850

**Published:** 2025-12-31

**Authors:** Hsin-Yu Fang, Helin Sera Şan, Fatma Akar, Sudenaz Fatma Oner, Ceren Şinikçi, Alexander Tyshkovskiy, Vadim Gladyshev, Perinur Bozaykut Eker

**Affiliations:** Tufts University, Boston, Massachusetts, United States; Acıbadem Mehmet Ali Aydınlar University, Istanbul, Istanbul, Turkey; Acıbadem Mehmet Ali Aydınlar University, Istanbul, Istanbul, Turkey; Acıbadem Mehmet Ali Aydınlar University, Istanbul, Istanbul, Turkey; Acıbadem Mehmet Ali Aydınlar University, Istanbul, Istanbul, Turkey; Harvard Medical School, Boston, Massachusetts, United States; Harvard Medical School, Boston, Massachusetts, United States; Tufts University, Boston, Massachusetts, United States

## Abstract

Aging is a predominant risk factor for many chronic diseases, yet shared mechanisms underlying effective longevity interventions remain poorly understood. To address this, we established a cross-species, multi-omics discovery platform that integrates conserved gene expression signatures to identify novel compounds with lifespan and healthspan extending potential. Here, we investigated whether top-ranked mTOR inhibitors could mitigate accelerated vascular aging in progeria, a premature aging disorder that manifests with early-onset vascular pathology. Induced pluripotent stem cell-derived vascular smooth muscle cells (iPSC-VSMCs) from progeria patients were treated with selected mTOR inhibitors and comprehensively assessed for cellular and molecular biomarkers of vascular aging. Cellular assays demonstrated a significant change of senescence phenotype through SA-β-gal and EdU incorporation, together with decreased inflammatory signaling, without induction of apoptosis. Drug treatment also lowered intracellular reactive oxygen species (ROS), in both replicatively senescent cells and cells subjected to biomechanical stress using a progeria-on-a-chip vascular model, underscoring their robustness across distinct aging-related stress conditions. Furthermore, drug treatment reduced both epigenetic and transcriptomic age, with transcriptomic improvements most prominently reflected in extracellular matrix organization and inflammation-related modules. These findings highlight the therapeutic potential of novel mTOR inhibitors identified through multi-omics screening in attenuating vascular aging in progeria and suggest broader strategies for preventing aging-associated vascular disease.

